# A Treg-Selective IL-2 Mutein Prevents the Formation of Factor VIII Inhibitors in Hemophilia Mice Treated With Factor VIII Gene Therapy

**DOI:** 10.3389/fimmu.2020.00638

**Published:** 2020-04-28

**Authors:** Alex C. Chen, Xiaohe Cai, Chong Li, Liliane Khoryati, Marc A. Gavin, Carol H. Miao

**Affiliations:** ^1^Center for Immunity and Immunotherapies, Seattle Children’s Research Institute, Seattle, WA, United States; ^2^Translational Research Program, Benaroya Research Institute, Seattle, WA, United States

**Keywords:** IL-2, hemophilia, factor VIII, inhibitors, Tregs, tolerance, gene therapy

## Abstract

Hemophilia A is a genetic disorder that results in the deficiency of functional factor VIII protein, which plays a key role in blood coagulation. Currently, the majority of hemophilia A patients are treated with repeated infusions of factor VIII protein. Approximately 30% of severe hemophilia A patients develop neutralizing antibodies to factor VIII (known as factor VIII inhibitors) due to treatment, rendering factor VIII protein infusions ineffective. Previously, mice receiving murine IL-2 complexed with α-murine IL-2 mAbs (JES6-1A12) showed a lack of factor VIII inhibitor formation after factor VIII treatment, which was associated with the proliferation and the activation of factor VIII-specific regulatory T cells (Tregs). In this paper, we evaluated if an Fc-fused mutated protein analog of mouse IL-2, named Fc.Mut24, engineered to selectively promote the expansion of Tregs *in vivo* can modulate factor VIII-specific immune responses. The mice received one intraperitoneal injection of Fc.Mut24. When the regulatory T cell population reached its highest frequency and peak activation, the mice received a hydrodynamic injection of factor VIII plasmid (day 4) followed by a second Fc.Mut24 dose (day 7). Peripheral blood was collected weekly. Flow cytometry was used to characterize the peripheral blood cell populations, while ELISA and Bethesda assays were used to assess the inhibitor concentrations and the functional titers in plasma. The activated partial thromboplastin time assay was used to assess the functional activities of factor VIII in blood. The mice receiving Fc.Mut24 showed a dramatic and transient increase in the population of activated Tregs after Fc.Mut24 injection. Factor VIII gene therapy *via* hydrodynamic injection resulted in high anti-factor VIII inhibitor concentrations in control PBS-injected mice, whereas the mice treated with Fc.Mut24 produced no inhibitors. Most significantly, there were no inhibitors generated after a second hydrodynamic injection of factor VIII plasmid administered at 19 weeks after the first injection in Fc.Mut24-treated mice. The mice receiving Fc.Mut24 maintained high levels of factor VIII activity throughout the experiment, while the control mice had the factor VIII activity dropped to undetectable levels a few weeks after the first factor VIII plasmid injection. Our data show that human therapies analogous to Fc.Mut24 could potentially provide a method to prevent inhibitor formation and induce long-term immune tolerance to factor VIII in hemophilia patients.

## Introduction

Hemophilia A (HemA) is a sex-linked recessive genetic disorder that results in a deficiency in factor VIII protein (FVIII), which is critical for blood coagulation. Currently, the majority of HemA patients undergo FVIII protein replacement therapy to acutely or prophylactically treat their condition (1). Unfortunately, approximately 30% of severe HemA patients develop alloantibodies to FVIII, often referred to as FVIII inhibitors, due to a lack of immune tolerance to FVIII. These inhibitors neutralize the FVIII activity and thus render conventional protein therapy ineffective.

To overcome the barriers to therapy caused by FVIII inhibitors, some patients undergo immune tolerance induction (ITI) (2), which can involve months or even years of treatment (3, 4). Even after a costly and long ITI regimen, only 70% of patients have successful outcomes (5, 6). More recently, the monoclonal bispecific antibody emicizumab has shown promise in providing an alternative to FVIII protein therapy for HemA patients with inhibitors (7, 8). However, repeated infusions over the patient’s lifetime are still required, and long-term safety needs to be evaluated. In addition, due to the growing potential of FVIII gene therapy for HemA patients (9–11), we believe that addressing the problem of FVIII inhibitors directly is still a very important pursuit.

Regulatory T cells (Tregs) are CD4^+^CD25^+^Foxp3^+^ T cells that are critical in establishing immune tolerance (12, 13). They also have important roles in coordinating a balanced response to foreign antigens (14, 15) and in the prevention of autoimmune diseases (16). Immunomodulating techniques involving the manipulation of Treg populations have shown promise in promoting FVIII tolerance (2, 17–19). Both the *in vivo* expansion of Tregs (20–23) and the adoptive transfer of *in vitro* expanded antigen-specific Tregs (18, 24), T cell receptor-engineered Tregs (25), or chimeric antigen receptor-engineered Tregs (26, 27) have proven efficacy in HemA mice.

Interleukin-2 (IL-2) is a cytokine that promotes the proliferation of T cells and is critical for the maturation and survival of Tregs (28, 29). IL-2 signals through a heterogeneous trimer receptor, consisting of the α (CD25), β (CD122), and γ (CD132) chains (30). Signaling occurs through the β and γ chains, while the α chain increases the affinity between IL-2 and the receptor complex 100-fold (31). Because the α chain is present in high quantities on the surface of Tregs, the Tregs are more responsive to low IL-2 concentrations in comparison to the effector T cells. As such, IL-2 selectively increases Treg survival and proliferation when administered *via* a low-dose regimen (32–34) or when complexed with an anti-IL-2 mAb (JES6-1A12) that increases the CD25 dependency for IL-2R signaling (20, 22).

High-dose recombinant human IL-2 (aldesleukin) was originally approved as a cancer immunotherapy due to its stimulatory activity on cancer-killing effector CD4^+^ and CD8^+^ T cells and NK cells (35, 36). More recently, chemically modified (37, 38) and computationally designed versions of IL-2 (39) have shown promise in increasing the effectiveness and decreasing the side effects associated with wild-type IL-2 treatment. With the newly appreciated role for IL-2 in Treg function, recent studies have explored low-dose IL-2 for the treatment of auto-inflammatory diseases through Treg enrichment (40, 41). While exploratory clinical studies have shown that low-dose IL-2 is generally well tolerated and that efficacy in resolving disease symptoms can occur, the possibility that Tregs are not adequately activated at the low doses required to avoid effector T cell responses raises concerns that a generally applicable dosing strategy will be difficult to define and may ultimately result in only moderate efficacy (42–44).

To overcome these limitations, mutational variants of IL-2—fused to Fc or IgG domains to increase half-life and exposure—have been developed with greater Treg selectivity due to a greater reliance on high CD25 expression for IL-2R signaling (45, 46). While the clinical testing of these molecules is just beginning, the general applicability, robustness, and durability of this approach should be more extensively explored with murine surrogates of experimental therapeutics. In this study, we utilized a highly Treg-selective mutated version of murine IL-2, referred to as Fc.Mut24 (47), to activate and increase the Treg population in HemA mice, followed by gene therapy to induce FVIII tolerance. An analysis of the peripheral blood serum from Fc.Mut24-treated mice showed the absence of FVIII inhibitors and the high levels of functional FVIII throughout the experiment. In contrast, the control mice quickly developed inhibitors and had the functional FVIII levels dropped to negligible levels early in the experiment. Tolerance to FVIII was maintained in the mice for the 6-month experiment duration, even after a second gene therapy challenge was administered.

## Materials and Methods

### Mice

All mice were kept in accordance with the National Institute of Health and Seattle Children’s Research Institute guidelines for animal care. The mice were housed in a specific pathogen-free facility. HemA mice of mixed 129/SV and BL6 genetic background were generated by the targeted disruption of exon 16 of the FVIII gene (48). The experiments started in 8- to 12-week-old mice.

### Immunomodulation With Fc.Mut24 and Gene Therapy of FVIII

The identification and characterization of Fc.Mut24 is described in the study of Khoryati et al. (47). Briefly, site-directed mutagenesis was performed on murine IL-2, which was then fused to an effector-functionless murine IgG2a Fc. Twenty-eight different IL-2 muteins were screened for efficacy, with Fc.Mut24 being selected for this study. The Fc.Mut24 protein was generated at Olympic Protein Technologies (Seattle, WA, United States). The experimental mice initially received 6 μg of Fc.Mut24 in 200 μl of phosphate-buffered saline (PBS) by intraperitoneal injection, while the control mice received 200 μl of plain PBS. FVIII gene therapy was administered *via* a hydrodynamic injection of 50 μg of FVIII plasmid (pBS-HCRHPI-FVIIIA) in PBS 4 days after the initial Fc.Mut24 injection. A second Fc.Mut24 injection of 3 μg Fc.Mut24 in 100 μl PBS for the experimental mice was performed at 7 days after the initial injection, while the control mice received 100 μl of plain PBS.

### Characterization of Peripheral Blood Mononuclear Cells and Splenocytes

Flow cytometry was used to characterize the peripheral blood mononuclear cells (PBMCs) and splenocytes. Peripheral blood was collected *via* retro-orbital bleeding. Splenocytes were collected from spleens that were homogenized by grinding between glass slides. The cells were stained with the following antibodies conjugated to fluorophores: Alexa Fluor 700- anti-mouse CD4 (BD Biosciences, San Jose, CA, United States), PE-Cy5- anti-mouse CD25 (eBioscience, San Diego, CA, United States), PE-CF594- anti-mouse Foxp3 (BD Biosciences), PE- anti-mouse CTLA-4 (eBioscience), FITC-anti-mouse Helios (Biolegend, San Diego, CA, United States), PE-anti-mouse CD11b (BD Biosciences), APC-Cy7 anti-mouse CD8a (BD Biosciences), and Alexa Fluor 700-anti-mouse B220 (eBioscience). The cells were fixed and permeabilized before staining with the eBioscience Foxp3/transcription factor staining buffer set. Flow cytometry was performed on a LSRII flow cytometer (BD Biosciences) and data were analyzed using FlowJo software (FlowJo LLC, Ashland, OR, United States). Gating strategy is included in the Supplementary Figure 1.

### FVIII Activity and Anti-FVIII Antibody Assays

Peripheral blood samples were collected from mice in 3.8% sodium citrate solution. Blood plasma was separated *via* centrifugation. FVIII activity was measured *via* activated partial thromboplastin time (aPTT) using a modified clotting assay with FVIII-deficient plasma (18). The anti-FVIII IgG1 concentration was measured *via* enzyme-linked immunosorbent assay (ELISA). The FVIII inhibitor concentration was measured *via* Bethesda assay.

### Treg Suppression Assay

CD4^+^ cells were isolated from the spleens of mice by magnetic activated cell sorting (Miltenyi Biotec, Auburn, CA, United States). The total CD4^+^ splenocytes from mice with high anti-FVIII antibody serum concentrations were used as responder T cells (Tresps). CD4^–^ splenocytes from naïve mice were irradiated and used as antigen-presenting cells (APCs). CD4^+^CD25^+^ cells were used as Tregs and were isolated by magnetic separation from naïve mice or mice tolerized *via* Fc.Mut24 and FVIII gene therapy. RPMI medium supplemented with 10% fetal bovine serum, 1% L-glutamine, 1% HEPES, 1% penicillin–streptomycin, 1% sodium pyruvate, 0.1% 2-mercaptoethanol, and murine IL-2 at 100 U/ml was used in all culture conditions. APCs were plated at 1.6 × 10^5^ cells per well, and Tresps were plated at 8 × 10^4^ cells per well. Tregs were plated at either 8 × 10^4^ or 4 × 10^4^ cells per well. The cells were stimulated with FVIII at 10 U/ml for 5 days. Proliferation of cells was quantified using the Click-iT Plus EdU flow cytometry assay kit (Invitrogen, Carlsbad, CA, United States). Then, 10 μM of EdU reagent was added to the culture media immediately after plating. Details on the analysis of the Treg-suppressive activity are described in the Supplementary Methods.

### Non-specific Challenge With Trinitrophenyl-Ficoll and Trinitrophenyl-Keyhole Limpet Hemocyanin

Fc.Mut24-treated mice (*n* = 3) and naïve mice (*n* = 3) were intraperitoneally immunized with 25 μg trinitrophenyl-Ficoll (TNP-Ficoll) and 100 μg trinitrophenyl-keyhole limpet hemocyanin (TNP-KLH) reconstituted in 100 μl PBS and then emulsified in an equal volume of complete Freund’s adjuvant at week 16. A second challenge was administered 3 weeks later with the same volume and concentrations. Serum was collected from the mice weekly and analyzed for anti-TNP antibodies by ELISA in plates coated with TNP-bovine serum albumin. IgG concentrations were detected using horseradish peroxidase-labeled anti-mouse IgG antibodies to develop the substrate solution.

### Statistical Analyses

All data are presented as means ± standard deviation. Statistical significance for single time points was calculated using the parametric Mann–Whitney *U* test due to the small sample sizes. Statistical significance between groups across multiple time points was calculated using repeated- measures ANOVA.

## Results

### Fc.Mut24 Increases and Activates the Treg Population *in vivo*

**FIGURE 1:**
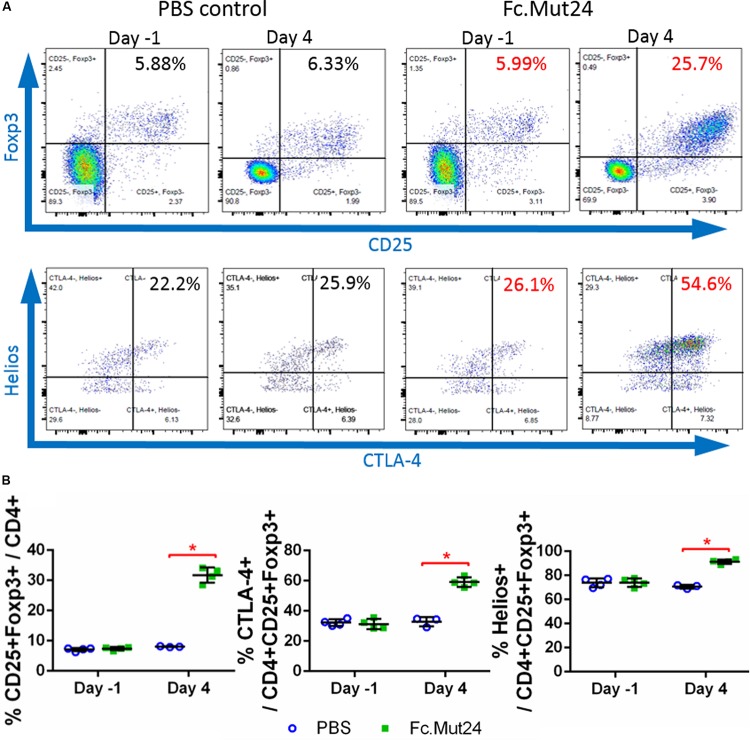
Analysis of changes in CD4^+^CD25^+^Foxp3^+^ Tregs by IL-2 Fc.Mut24 treatment. Fc.Mut24 was administered to HemA mice (*n* = 4) and lymphocyte populations were compared to mice receiving phosphate-buffered saline (PBS) injections (*n* = 3). **(A)** Representative flow cytometry plots of CD4^+^ peripheral blood lymphocytes to determine the percentage of the Treg population, defined by double-positive CD25 and Foxp3 expression (top row). CD25^+^Foxp3^+^ Tregs were gated to determine the activation percentage, defined by CTLA-4 expression (bottom row). **(B)** Comparison of percentages of Tregs (left), activated Tregs (middle), and Helios^+^ Tregs (right) between PBS- and Fc.Mut24-treated mice on Day 1 and Day 4. The experiments were repeated three times with no significant variation. The data are presented as means with standard deviation. The *p*-values were calculated at individual time points by non-parametric Mann–Whitney *U* test (*p < 0.05).

We assessed the ability of Fc.Mut24 to increase and activate Tregs in HemA mice by intraperitoneally injecting 6 μg of Fc.Mut24 diluted in 200 μl PBS. PBMCs isolated from Fc.Mut24-treated mice and PBS-treated control mice were analyzed by flow cytometry and stained by Treg markers, CD4, CD25, and Foxp3. The CD4^+^CD25^+^Foxp3^+^ Treg population in the control mice remained at ∼7.5% of the total CD4^+^ cells between 1 day before treatment (day -1) and 4 days post-treatment (day 4). The experimental mice showed an approximately fourfold increase (from 7.3 to 31.7%) in Treg population from day -1 to day 4. Treg activation, defined by CTLA-4 positive staining, was doubled by day 4, increasing from 31 to 59% (Figure 1). Treg activation remained unchanged in the PBS-treated control mice. Interestingly, most of the expanded Tregs in the Fc.Mut24-treated mice were Helios^+^ cells, increasing from 74 to 92% of the Treg population, whereas the percentage of Helios^+^ Tregs remained the same in the PBS-treated control mice. Helios^+^ Tregs have been described to be predominantly derived from the thymus and are considered to be more stable and highly suppressive compared to Helios^–^ Tregs (49).

### Fc.Mut24 Treatment in Conjunction With FVIII Gene Therapy Prevents Inhibitor Formulation and Maintains FVIII Clotting Activity

**FIGURE 2:**
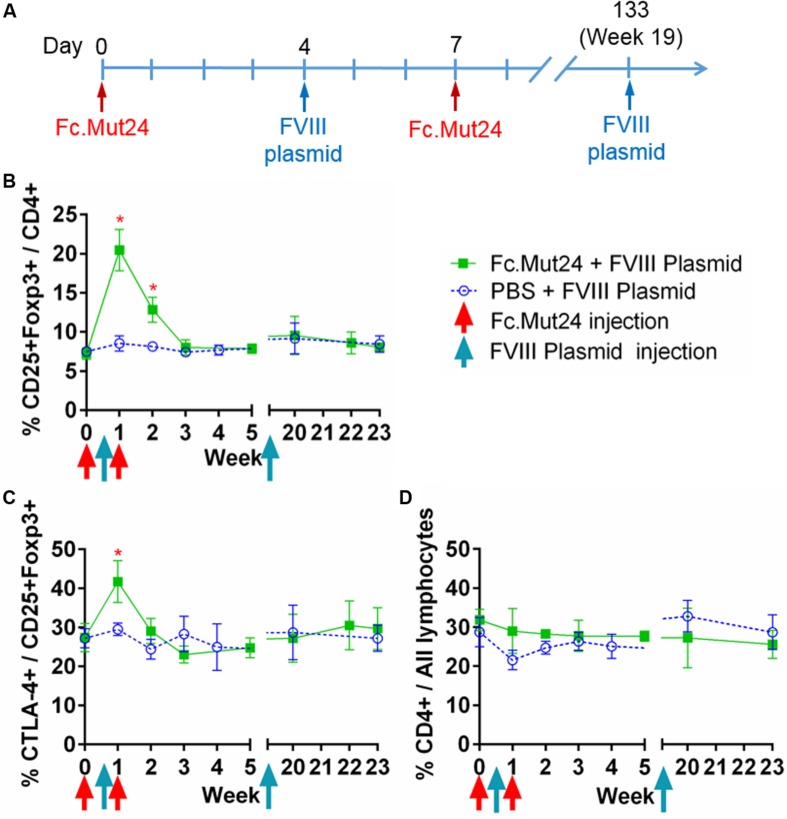
The effect of Fc.Mut24 on the peripheral blood lymphocytes of mice receiving Fc.Mut24 (*n* = 4) or PBS (*n* = 3) combined with FVIII plasmid gene therapy. The peripheral blood lymphocytes were characterized *via* flow cytometry over 23 weeks. The lymphocytes were first gated on live cells and then CD4 to determine the helper T-cell population. **(A)** The Fc.Mut24 and FVIII plasmid dosing schedule. Six micrograms of Fc.Mut24 was injected intraperitoneally into the experimental mice on day 0 and 3 μg was injected on day 7. The FVIII plasmid was administered hydrodynamically to all mice on day 4. **(B)** The percentage of Treg population was defined by CD25^+^Foxp3^+^ cells out of the total CD4^+^ population. Days 7 and 14: *p < 0.05. **(C)** The activation of Tregs was measured by percentage, showing the high CTLA-4 expression. Day 7: *p* < 0.05. **(D)** The CD4^+^ population as a percentage of total lymphocytes over the 23 weeks of the experiment. The experiments were repeated three times with no significant variation. The data are presented as means with standard deviation. The *p*-values were calculated at individual time points by non-parametric Mann–Whitney *U* test.

After confirming the efficacy of Fc.Mut24 in activating Tregs and stimulating their proliferation, we performed a gene therapy experiment in conjunction with Fc.Mut24 treatment as described in Figure 2A. HemA mice were treated with 6 μg Fc.Mut24 or vehicle control on day 0 as before. On day 4, all mice received a hydrodynamic injection of 50 μg FVIII plasmid. On day 7, the mice received a second 3-μg injection of Fc.Mut24 in 200 μl PBS (experimental group) or vehicle (control group).

Peripheral blood mononuclear cells from the treated mice were isolated and analyzed over 7 weeks following the Fc.Mut24 treatment. Peak Treg population and activation percentages in the Fc.Mut24-treated mice were observed between day 4 and day 7, which then dropped significantly by day 14. The Treg population returned to baseline levels by day 21, while Treg activation did not differ significantly from the control mice by day 14 (Figures 2B,C). The percentage of total CD4^+^ cells in relation to all PBMCs did not change for either group throughout the experiment, indicating that Fc.Mut24 selectively enriched the Treg population (Figure 2D). FVIII plasmid was administered on day 4 to coincide with peak Treg population and activation.

**FIGURE 3:**
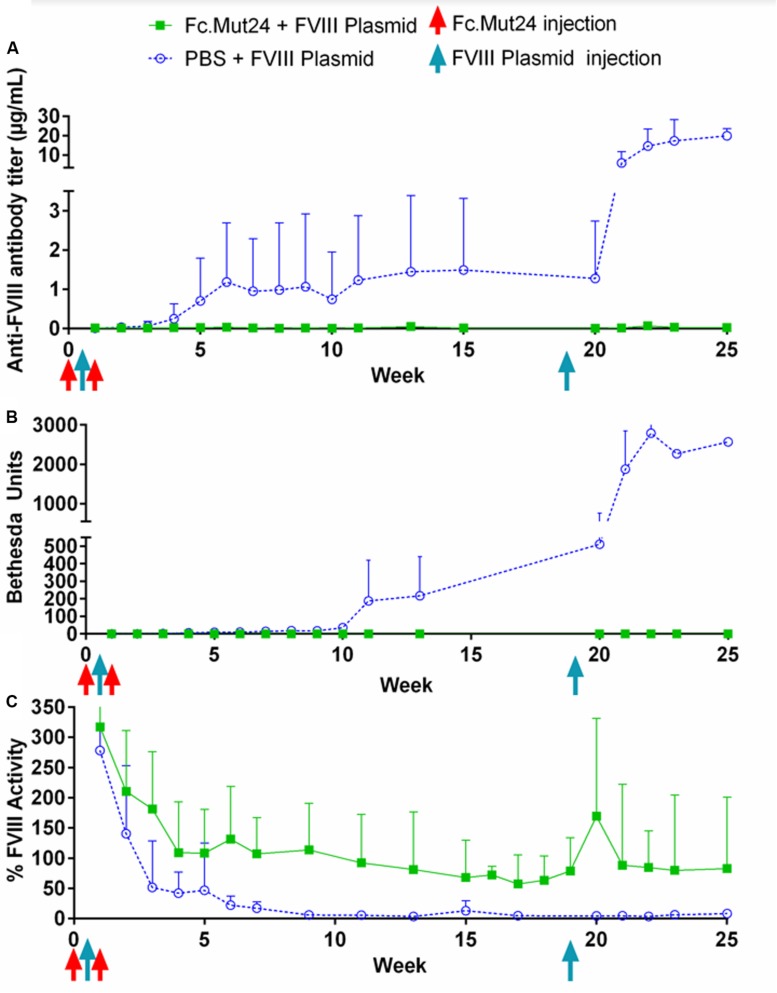
Analysis of peripheral blood plasma of mice receiving Fc.Mut24 or PBS. Peripheral blood was collected *via* retro-orbital bleeding in sodium citrate and spun at 500*g* for 5 min to separate plasma from cells. **(A)** ELISA was performed to measure anti-FVIII antibody levels in peripheral blood plasma using mouse whole IgG as control. *p* = 0.021 between Fc.Mut24-treated (*n* = 8) and PBS-treated (*n* = 4) groups. **(B)** Bethesda assay was performed on peripheral blood plasma. Serial dilutions were performed on plasma samples that showed high levels of inhibitor to obtain accurate inhibitor titers. *p* = 0.0092 between Fc.Mut24- and PBS-treated groups at multiple time points. **(C)** Factor VIII activity was measured in peripheral blood plasma *via* activated partial thromboplastin time assay relative to normal human plasma controls. *p* = 0.0014 between Fc.Mut24- and PBS-treated groups at multiple time points. The experiments were repeated twice with no significant variation. The data are presented as means with standard deviation. The *p*-values were calculated for multiple time points between groups using repeated-measures ANOVA.

Plasma was collected from Fc.Mut24 + FVIII plasmid-treated and PBS + FVIII plasmid-treated mice and analyzed weekly to quantify the anti-FVIII antibody levels by ELISA and the anti-FVIII functional inhibitor titers by Bethesda assay. These analyses showed that the control mice receiving PBS had 0.25 μg/ml of anti-FVIII antibodies by week 4 (Figure 3A), which increased to 1 μg/ml by week 7, which was when they began to plateau. FVIII inhibitor levels were detected at 6.5 BU by week 4 and then increased gradually to ∼200 BU by week 11, where they remained relatively consistent until the secondary challenge (Figure 3B). The experimental mice receiving Fc.Mut24 in conjunction with gene therapy showed zero or negligible levels of both anti-FVIII antibodies and FVIII inhibitors throughout the experiment.

FVIII activity was measured *via* a modified aPTT assay using normal human pooled plasma as controls. Both groups of mice showed very high levels (>200%) of FVIII activity 1 week after the initial FVIII plasmid gene therapy. These levels rapidly dropped to below 50% by week 3 in the control mice, which further dropped to undetectable or negligible levels by week 10. The experimental mice showed a gradual decrease in FVIII activity for the first 4 weeks of the experiment to ∼100%. This activity level remained relatively stable but decreased to about 70% by week 19 (Figure 3C).

### Long-Term FVIII Activity and FVIII Tolerance Are Maintained in Mice Treated With Fc.Mut24 After the Secondary FVIII Gene Therapy

To investigate the ability of Fc.Mut24 treatment to induce long-term FVIII tolerance, we performed a second FVIII gene therapy challenge in all mice. Thus, 50 μg of FVIII plasmid was injected hydrodynamically on week 19. The control mice showed a significant increase in anti-FVIII antibodies after the secondary challenge, from 1.5 μg/ml at week 15 up to 6 μg/ml at week 21 and to 20 μg/ml by week 25, and continued to show undetectable levels of FVIII. The elevation of the immune responses was also reflected in the inhibitor titers, increasing from 200 to 1,000 BU from week 13 to week 23. In contrast, in Fc.Mut24-treated mice, we observed an increase in FVIII activity to 170% at 1 week after the secondary challenge, which then decreased and plateaued at ∼80% activity within 2 weeks (Figure 3C). The Fc.Mut24-treated mice maintained low or negligible levels of anti-FVIII antibodies and inhibitors for the remaining duration of the experiment (Figures 3A,B).

### Fc.Mut24 + FVIII Gene Therapy Combination Treatment Increases the FVIII-Specific Immunosuppressive Capacity of Splenic Tregs

**FIGURE 4:**
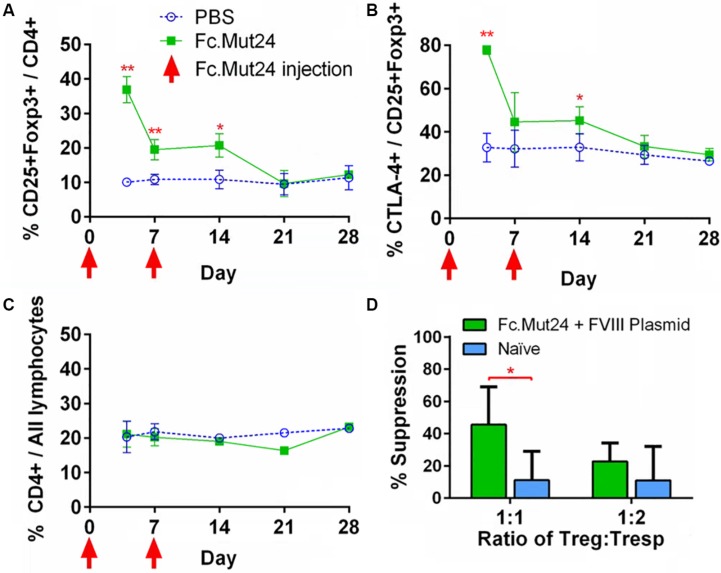
Characterization and functional evaluation of CD4^+^ splenocytes in Fc.Mut24-treated and FVIII plasmid gene therapy-treated mice. **(A–C)** Splenocytes were isolated from mice receiving Fc.Mut24 (*n* = 3) or PBS (*n* = 3) treatment and characterized *via* flow cytometry for comparison with peripheral blood lymphocytes. The experiments were repeated three times without significant variation. The data are presented as means with standard deviation (*p < 0.05, **p < 0.01). The *p*-values were calculated at individual time points by non-parametric Mann–Whitney *U* test. **(D)** A suppression assay was performed using CD4^+^CD25^+^ Tregs isolated on Day 50 after an initial treatment from the spleens of mice receiving Fc.Mut24 and FVIII plasmid or from naïve mice (*n* = 6 for all groups). Tregs were cultured *in vitro* with CD4^+^ cells isolated from the spleens of inhibitor-positive hemophilia A mice as well as irradiated antigen presenting cells. FVIII was added at 10 U/ml in culture media for 4 days. FVIII-specific cell proliferation was measured by an EdU incorporation assay and analyzed *via* flow cytometry. The suppression of CD4^+^ cell proliferation was compared to CD4^+^ cell proliferation in the absence of Tregs. The data are presented as means with standard deviation from two separate experiments (*p < 0.05).

To characterize the splenocytes of mice treated with Fc.Mut24, spleens were isolated, homogenized, and lysed with ACK buffer. The resulting splenocytes were stained and characterized by flow cytometry, as described above. The Treg population and activation percentages in the spleen reflected the trends observed in the PBMCs, which peaked at around day 4, significantly dropped by day 7, and returned to baseline levels by day 21 (Figures 4A,B). The overall CD4^+^ population did not show any significant changes, again indicating that Fc.Mut24 selectively enriched the Treg population (Figure 4C). To assess the functional capacity of Tregs from the treated mice, spleens were harvested from the experimental mice at day 50 and isolated Tregs were evaluated with an *in vitro* suppression assay. The Tregs from mice receiving Fc.Mut24 + FVIII gene therapy were isolated and cultured with irradiated CD4^–^ cells as APCs, Tresps from mice with high FVIII inhibitor titers, and FVIII protein for 5 days. Their efficacy in suppressing Tresp proliferation was compared to the suppressive activity of the Tregs isolated from naïve mice. When cultured at a ratio of 1:1 Treg/Tresp, we observed an increase in the suppressive potential of Tregs isolated from Fc.Mut24-treated mice (Supplementary Figure 2). Fc.Mut24 Tregs were able to suppress Tresp proliferation by 45.6%, while naïve Tregs only suppressed 11.2% of Tresp proliferation, an approximately fourfold increase in suppressive potential (Figure 4D). When the Treg/Tresp ratio was changed to 1:2, the difference in suppressive potential was only 28.2 to 11%, an increase of only 2.5-fold. These results indicate that the Tregs isolated from Fc.Mut24-treated mice have a functional population of Tregs that are specific to FVIII that persists for several weeks. These Tregs show the potential to suppress an immune response initiated by FVIII-specific Tresps.

### Fc.Mut24 Treatment Does Not Alter Antigen-Presenting Cell or CD8^+^ T Cell Frequencies

**FIGURE 5:**
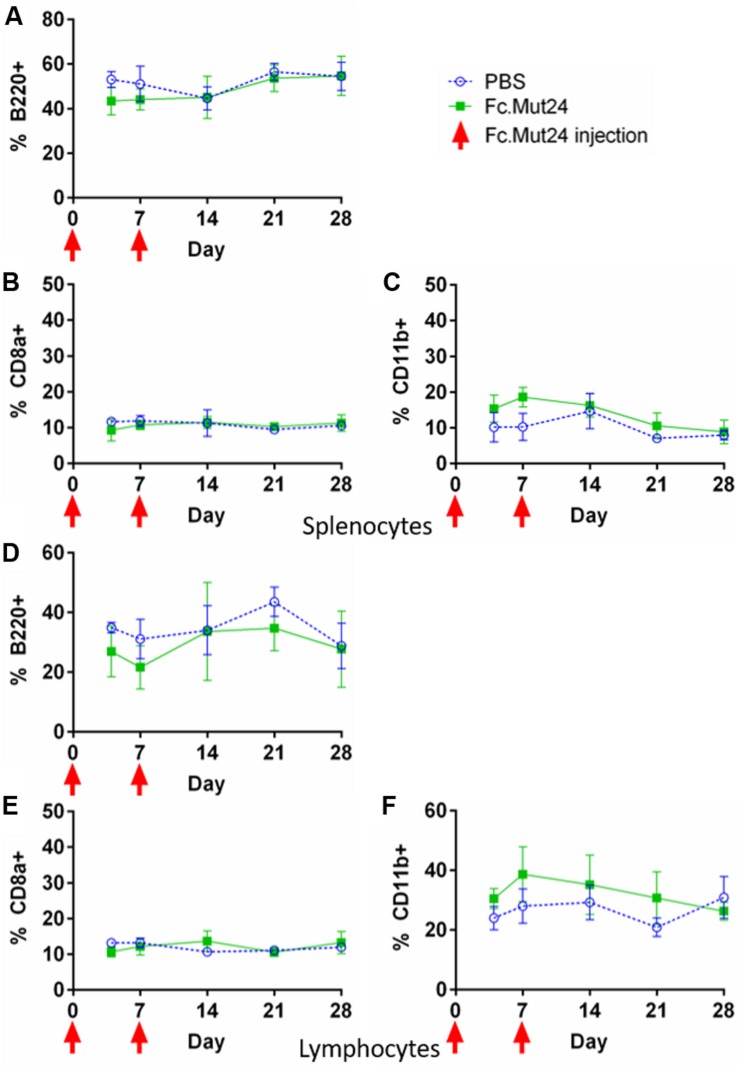
Characterization of B220^+^, CD8a^+^, and CD11b^+^ cells in the spleen and the peripheral blood of Fc.Mut24-treated and FVIII plasmid gene therapy-treated mice. The mice treated with Fc.Mut24 (*n* = 3) or PBS (*n* = 3) in conjunction with FVIII plasmid gene therapy were sacrificed at separate time points, and their spleens **(A–C)** and peripheral blood **(D–F)** were collected for analysis by flow cytometry. B220 was used as a marker for B cells **(A,D)**, CD8a as a marker for cytotoxic T cells **(B,E)**, and CD11b as a marker for myeloid cells **(C,F)**. All percentages are calculated from total lymphocytes from peripheral blood or total splenocytes from spleens. The experiments were repeated at least twice without significant variation. The data are presented as means with standard deviation. *p* > 0.05 at all time points. The *p*-values were calculated at individual time points by non-parametric Mann–Whitney *U* test.

In order to characterize different cell populations in the spleen during Fc.Mut24 treatment, we analyzed splenocytes and PBMCs *via* flow cytometry as described above. B220 was used as a marker for B cells, myeloid cells were defined as CD11b^+^ cells, and cytotoxic T cells were defined as CD8a^+^ cells. During the Fc.Mut24 treatment, we observed small differences between the experimental and the control groups for B220^+^ and CD11b^+^ cells. The Fc.Mut24-treated mice seemed to have a slightly lower B220^+^ population than the control mice on day 4 and day 7, which then returned to comparable levels by day 14 (Figures 5A,D). The Fc.Mut24-treated mice also seemed to have a slightly higher population of CD11b^+^ cells from day 4 to day 14 (Figures 5C,F). However, none of these differences was statistically significant at any time point (*p* > 0.05). There were no differences seen in the CD8a^+^ cell populations between the control and the experimental groups at any time point (Figures 5B,E). Although NK cells have a strong response to wild-type IL-2, we did not characterize NK cell populations specifically because Fc.Mut24 was shown to have a minimal effect on NK cell populations *in vivo* at the doses used in these experiments (47).

### Unrelated Antigen Challenge Shows the Immune Competence of Fc.Mut24-Treated Mice

**FIGURE 6:**
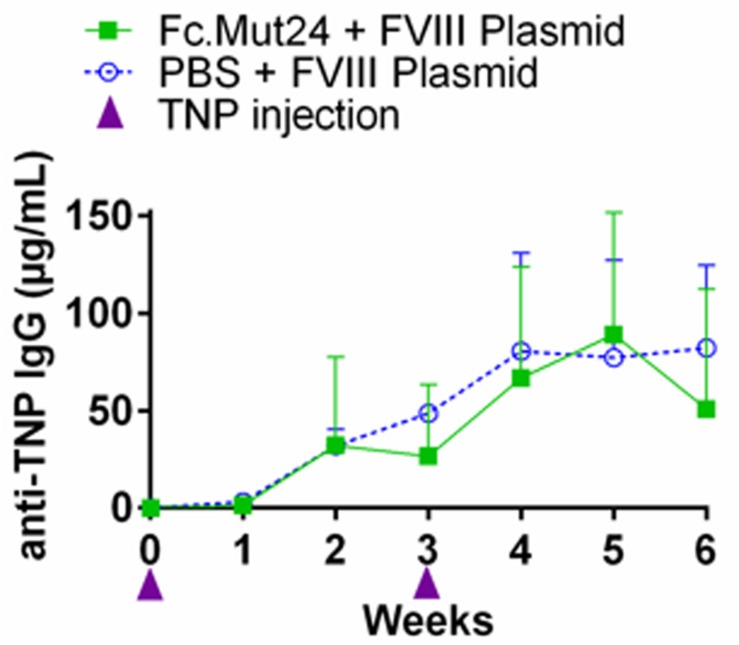
Evaluation of the immune competence of the Fc.Mut24-treated and the FVIII plasmid gene therapy-treated mice. Trinitrophenyl (TNP) antigen challenge was performed to evaluate if the mice treated with Fc.Mut24 and FVIII plasmid gene therapy remain immune-competent to other unrelated antigens. At 16 and 19 weeks after the initial Fc.Mut24 treatment, the experimental (*n* = 3) and the control naïve hemophilia A mice (*n* = 3) were injected with TNP-keyhole limpet hemocyanin and TNP-Ficoll emulsified in CFA to induce a non-specific immune response. The anti-TNP IgG concentrations were measured *via* ELISA (*p* = 0.66). The *p*-values were calculated at multiple time points between groups using repeated-measures ANOVA.

While Fc.Mut24 treatment induced FVIII tolerance when FVIII gene therapy was administered during Treg enrichment, we wanted to determine if the suppression of antibody responses against other antigens persisted in treated mice after the Tregs returned to baseline frequencies. To evaluate the immune competence of mice receiving Fc.Mut24, an unrelated antigen challenge was performed using TNP-KLH and TNP-Ficoll. At week 16 post-gene therapy, three mice receiving Fc.Mut24 + FVIII plasmid that showed high FVIII functional activity were injected with TNP-KLH and TNP-Ficoll in complete Freund’s adjuvant. A second challenge with the same emulsified antigens was performed at week 19. Three naïve mice receiving the same antigen challenge were used as a control group. Peripheral blood serum was collected and ELISA was used to measure the TNP IgG levels to assess the immune response of the experimental mice compared to the naïve controls. The antibody titers showed no significant difference between the Fc.Mut24-treated mice and the naïve mice at any time point. The anti-TNP IgG levels gradually increased to ∼80 μg/ml in both groups by week 5 after the initial challenge (Figure 6). At no time point was there a statistical significance between the experimental mice and the naïve mice, indicating that the transient Fc.Mut24 treatment did not permanently compromise the murine immune system.

## Discussion

The alloimmune response to FVIII treatment is a complication that occurs in a significant number of severe HemA patients. CD4^+^CD25^+^Foxp3^+^ Tregs have been demonstrated to be critical in the suppression of alloimmune and autoimmune responses (50, 51). Some approaches to prevent the development of FVIII inhibitors require *ex vivo* expansion and manipulation of Tregs, which are then adoptively transferred to suppress the immune response (18, 24). These techniques require the isolation, expansion, and adoptive transfer of FVIII-specific Tregs, an inefficient and time-consuming process that risks disease exacerbation due to Treg dedifferentiation into effector T cells. Other techniques that have shown efficacy involve the *in vivo* expansion of Tregs using murine IL-2 complexed with a specific anti-murine IL-2 antibody (clone JES6-1A12) (20, 22).

This study explores a more robust Treg enrichment method with direct applicability to the design of human trials using Fc.Mut24 (47), a murine surrogate for half-life-extended human IL-2 muteins designed specifically to increase and activate the Treg population while having a minimal effect on the rest of the immune system (45, 46). In brief, Fc.Mut24 contains mutations in the region that interacts with the CD122 portion of the IL-2 receptor, increasing reliance on binding with CD25 for binding and subsequent cell signaling (Supplementary Figure 3). This results in selective signaling in cells with CD25^*high*^ expression and low or non-existent signaling in CD25^–^ cells.

The preliminary testing of Fc.Mut24 showed that the murine peripheral blood Treg population increased at least fourfold on day 4 after one intraperitoneal injection, which was also observed in the spleen Treg population. Previously, the IL-2/IL-2 antibody complex required three injections on three consecutive days in order to achieve a high expansion of Tregs *in vivo* (20, 22). The percent of CTLA-4^+^-activated Tregs after Fc.Mut24 injection also increased by twofold on day 4. These data encouraged us to perform FVIII gene therapy *via* the hydrodynamic injection of FVIII plasmid on day 4. A second lower-dose injection of Fc.Mut24 performed on day 7 was performed to maintain a relatively high Treg percentage during the initial expression of FVIII protein. We hypothesized that a high population of activated Tregs during the early timeframe of FVIII expression would prevent FVIII inhibitor formation by suppressing helper T cell function. Both Treg percentage and activation returned to normal levels by day 21.

The analysis of peripheral blood plasma showed that our dosage schedule of Fc.Mut24 and FVIII gene therapy was highly effective in preventing the formation of FVIII inhibitors. The Fc.Mut24-treated mice showed no anti-FVIII antibodies at any time throughout the course of the experiment, even after a second FVIII challenge on week 19. In addition, the analysis of functional FVIII activity in blood plasma showed that FVIII activity remained high in Fc.Mut24 mice as well. This was in stark contrast with that in the control mice, which had significant levels of anti-FVIII antibodies by week 5 that increased greatly after the secondary FVIII challenge. The FVIII activity in the control mice also dropped to negligible levels by week 10. An *in vitro* evaluation of Tregs from Fc.Mut24-treated mice demonstrated an increased suppressive response compared to the Tregs from naïve mice when cultured in the presence of FVIII protein. This leads us to believe that FVIII-specific Tregs were present in significant numbers in the Fc.Mut24-treated mice, which resulted in the tolerance of FVIII protein *in vivo*. It will be interesting to compare the effectiveness of Fc.Mut24 to a wild-type Fc.IL-2 treatment in future work.

Due to its mechanism of action, the Fc.IL-2 mutein approach may have increased safety when compared to other immunomodulating techniques. Other techniques involve agents such as rapamycin (21), cyclophosphamide (52), and rituximab (53, 54), which can have serious side effects. In Fc.Mut24-treated mice, no significant variations were observed in CD4^+^ T cell populations, as well as other cellular compartments including B cells, myeloid cells, and CD8^+^ T cells, in both the blood and the spleen as examined by flow cytometry analysis at various time points, indicating that Fc.Mut24 did not have a deleterious or significant stimulatory effect toward other cell types (Supplementary Figure 4). NK cells, which also respond to IL-2, were shown by *Khoryati et al*. (47) to be unaffected following the treatment of Fc.Mut24 at varying dosages in healthy and type-1 diabetic B6 mice. It is also expected that there will not be significant changes in NK cells following Fc.Mut24 treatment in HemA mice. However, this will need to be verified in future studies. It is also interesting to note that most of the expanded Tregs in Fc.Mut24 mice are Helios^+^. Helios^+^ Tregs have been described to be derived from the thymus (55) and are phenotypically more stable, suppressive, and activated compared to Helios^–^ Tregs (56). In addition, due to the increased reliance on high CD25 expression, Fc.Mut24’s specificity and potency in promoting Treg proliferation and activation is high, which means that Treg specificity is retained at high doses and that immune tolerance can be sustained with infrequent dosing compared to other IL-2 therapies in HemA mice. This was also seen in the study in type-1 diabetic B6 mouse model (47). This increased safety is also reflected in our unrelated antigen challenges, which showed that the immune system of the treated mice was not compromised.

Our experiments solely focused on the prevention of FVIII inhibitor formation and preservation of FVIII functional activity in HemA mice before receiving FVIII gene therapy. Current immune tolerance induction techniques for patients with preexisting inhibitors have high cost and with only 70% success rate (57), which call for new approaches to treat patients with FVIII inhibitors. The use of half-life-extended IL-2 muteins, perhaps in combination with other techniques, could provide a more effective and faster method of inducing tolerance in these patients. Anti-human IL-2mAbs have also been developed to either increase (58) or decrease (59) Treg specificity and tested for applications in diabetes or oncology, respectively. These antibodies are in the clinical trial phase at the time of publication, demonstrating that IL-2 Treg specificity approaches are viable in human patients. In addition, molecules with very similar structure and activity to Fc.Mut24 are currently in clinical trials for auto-inflammatory diseases; thus, our findings may be readily translated to clinical exploration by combining current and emerging FVIII replacement strategies with Treg-selective human IL-2 muteins (45, 46).

Potential directions for future research could include the adoptive transfer of Tregs from Fc.Mut24-treated mice to recipient HemA mice with preexisting inhibitors. If Fc.Mut24 treatment alone does not provide an avenue to induce FVIII tolerance, perhaps the infectious tolerance mechanism provided by FVIII-specific Tregs from donor mice would be able to (24, 60, 61). Previous experiments performed in this lab have shown similar effectiveness in preventing inhibitor formation using the IL-2/IL-2 antibody complex (20) and low-dose IL-2 using the same hydrodynamic gene therapy and mouse strain; a side-by-side comparison experiment could be performed to directly compare the effectiveness of these treatment methods. In addition, the use of half-life-extended IL-2 muteins and antigen therapy could also be applicable to other immune-related diseases that require the tolerance promoted by activated Tregs, such as organ transplant rejection (62, 63) and autoimmune and allergic diseases where the antigens promoting the immune-mediated pathology are known (64–67).

A limitation of this study is that we only used hydrodynamic injections as a means of gene therapy, which is not a feasible method of gene delivery for human patients at the time of this article’s publication. The delivery of the FVIII gene in humans would likely require other approaches, such as AAV vectors (9, 11) or lentiviral vectors (68, 69). However, these methods add another dimension of complexity in administering FVIII gene therapy, while hydrodynamic injection is straightforward and reproducible in a murine model. Hydrodynamic injections can also be consistently repeated at later time points for a secondary challenge, while viral vectors may induce anti-vector immune responses, reducing the effectiveness of a secondary challenge. Ultimately, hydrodynamic injection provides a method of gene therapy that reduces the likelihood of confounding factors in our studies, which focus on the reduction of inhibitors to FVIII. In addition, although the majority of patients currently receive FVIII protein as treatment, the future of hemophilia treatment is moving toward gene therapy approaches or synthetic biologicals such as emicizumab. This study focuses on addressing inhibitor formation following gene therapy.

In this study, it has been demonstrated that FVIII tolerance can be achieved in a murine model by using an engineered analog of IL-2, Fc.Mut24, in conjunction with FVIII gene therapy. Currently, many forms of hemophilia gene therapy are in development and appear to be the future of hemophilia treatment (10, 11). Thus, we believe that our use of FVIII plasmid gene therapy, rather than the use of FVIII protein injections, is a relevant test model for promoting FVIII tolerance following gene therapy. It would also be significant to evaluate this method combined with other gene delivery methods, such as viral vector-mediated gene therapy, in future studies. Compared to ITI and *ex vivo* cell therapy techniques, the *in vivo* expansion and activation of Tregs may potentially be a safer, more effective, and less costly approach to achieving FVIII tolerance for hemophilia patients. Corroborative experiments in non-naïve HemA models and other gene therapy techniques could support moving this approach forward, with eventual applications in human clinical trials.

## Data Availability Statement

The raw data supporting the conclusions of this article will be made available by the authors, without undue reservation, to any qualified researcher.

## Ethics Statement

The animal study was reviewed and approved by IACUC, Seattle Children’s Research Institute.

## Author Contributions

AC designed and performed the experiments, analyzed the results, and wrote and edited the manuscript. XC and CL performed the experiments. LK and MG designed, screened, and provided the Fc.Mut24. MG assisted in the experimental design and edited the manuscript. CM conceived the experiments, supervised the project, and wrote and edited the manuscript.

## Conflict of Interest

The authors declare that the research was conducted in the absence of any commercial or financial relationships that could be construed as a potential conflict of interest.
