# Supplementary material for: A Treg-Selective IL-2 Mutein Prevents the Formation of Factor VIII Inhibitors in Hemophilia Mice Treated With Factor VIII Gene Therapy
Source: Front Immunol. 2020 Apr 28;11:638. doi: 10.3389/fimmu.2020.00638 (PMC7198749; doi:10.3389/fimmu.2020.00638)
Supplement: Supplementary file 1 [file Data_Sheet_1.DOCX]

**Supplementary Materials**

**Methods**

**Flow cytometry gating strategy**

All cells were first gated on a side versus forward scatter plot to exclude dead cells and debris. No viability stain was used. For Tregs, cells were first gated on the CD4^+^ marker and then plotted on Foxp3 and CD25 axes. CD4^+^ cells expressing Foxp3^+^ and CD25^+^ markers were defined as Tregs. Treg expression of CTLA-4 and Helios was gated using total lymphocytes as threshold values (Supplementary Figure 1). Gating for CD8a, CD11b, and B220 were done on total lymphocytes after excluding dead cells and debris on a side versus forward scatter plot.

**Suppression assay calculations**

FVIII-specific suppression assay was performed using the Click-iT Plus EdU flow cytometry assay kit (Invitrogen, Carlsbad, CA) as described in the Methods. The EdU proliferation assay provides a very sensitive and reproducible way of detecting low levels of proliferation. This is needed due to the low number of FVIII specific Tregs in Fc.Mut24 treated mice, and the low number of FVIII specific T responder cells in inhibitor mice. Dye based proliferation assays, such as CFSE and VPD450, do not provide enough sensitivity to detect very low percentages of cell proliferation.

During analysis, flow cytometry will result in the loss of some cells during the staining and acquisition processes. Due to this, the calculations of proliferation and suppression cannot be assessed directly by using cell counts. Percentages of proliferation also cannot be used without calibration to total cells in each condition, as different conditions will have different total cell numbers. In addition, Tregs from naïve mice may have different proliferation rates compared to Tregs from Fc.Mut24 treated mice. To accurately calculate the relative proliferation and suppression of different conditions, different controls were incorporated into the experimental design, including Tregs from Fc.Mut24 treated mice only and Tregs from naïve mice only, to accurately subtract background proliferation that results from Treg proliferation. Since irradiated APCs could not proliferate and were present at the same seeding density in all conditions, they were excluded from all calculations.

Background proliferation from Tregs is first calibrated using the seeded cell ratios for each condition. This calibrated background proliferation is then subtracted from the total CD4^+^ cell proliferation, giving us inhibitor CD4^+^ cell proliferation. This needs to be done separately for conditions with Fc.Mut24 Tregs and naïve Tregs, due to these cells coming from different animals. The percentage suppression is then calculated by the equation:

**100 * [(inhibitor CD4^+^ proliferation without Tregs) – (inhibitor CD4^+^ proliferation with Tregs)]**

**/ [inhibitor CD4^+^ proliferation without Tregs]**

We can then compare the percentage suppression between the Fc.Mut24 and naïve Treg groups for both the 1:1 and 1:2 ratios.

**Supplementary Figures**


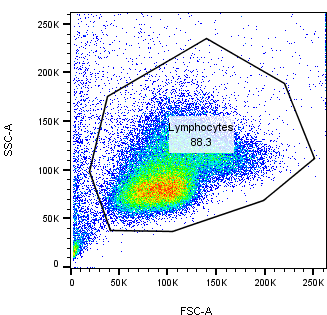

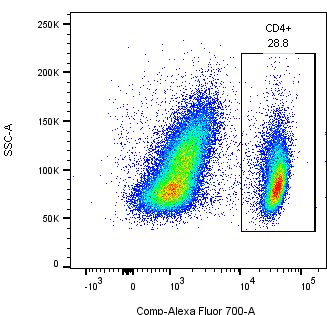

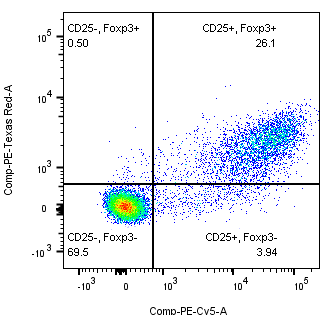

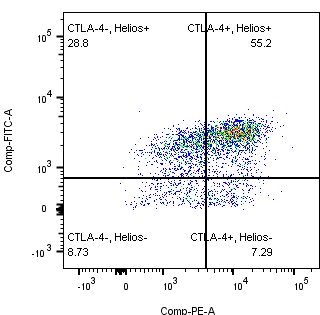


**C**

**D**

**B**

**A**


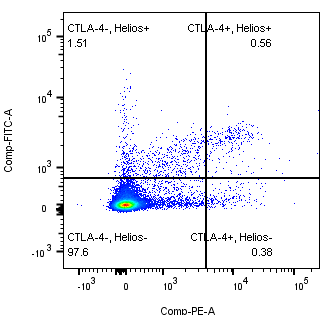


**E**

**Supplementary Figure 1 | Gating strategy for peripheral blood mononuclear cell analysis. (A)** Lymphocytes were gated on FSC and SSC scatter plot by excluding cell debris. **(B)** CD4^+^ cells gated on lymphocytes using AlexaFluor 700. **(C)** CD25^+^Foxp3^+^ Treg quadrant gated on CD4^+^ cells using PE-Cy5 and PE-TexasRed, respectively. **(D)** CD4^+^CD25^+^Foxp3^+^ were gated on CTLA-4 and Helios using PE and FITC, respectively. **(E)** CTLA-4 and Helios quadrant thresholds were determined using total lymphocytes.

**A**

**
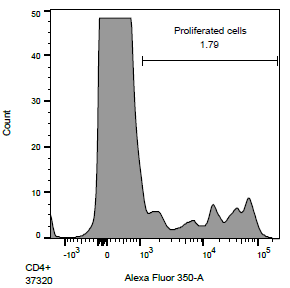
**

Tregs from Fc.Mut24 treated mice

**B**

**
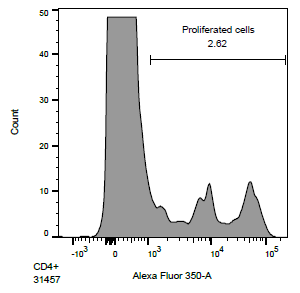
**

1:1 1:2

Treg : Tresp

Tregs from naive mice

**Supplementary Figure 2 | Flow cytometry plots of cell proliferation during the suppressive assay.** Representative plots of CD4^+^ cell proliferation indicated by the EdU assay. Tregs isolated from Fc.Mut24 treated experimental mice **(A)** or naïve mice **(B)**, at 1:1 (left) and 1:2 (right) ratios relative to Tresps. EdU incorporation into proliferating cells is measured on the Alexa Fluor 350 channel.


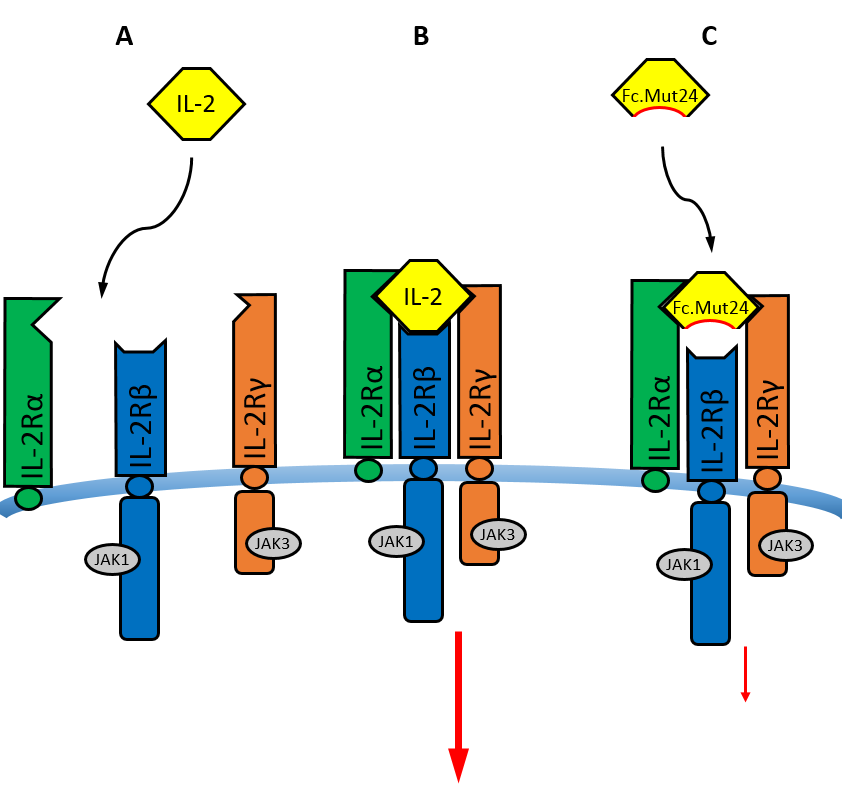


**Supplementary Figure 3 | Schematic of the interactions of IL-2 or Fc.Mut24 with the IL-2 Receptor complex on the cell surface.** (**A)** Human IL-2R is composed of three chains including IL-2Rα, β, and γ chains, which are dispersed on the cell surface. **(B)** The three receptor chains of IL-2R form a trimer upon binding with IL-2. Signaling occurs through the β and γ chains, while the α chain greatly increases binding affinity between IL-2 and the IL-2R trimer. **(C)** Fc.Mut24 has a mutation in the region that interacts with the β chain, resulting in decreased signaling through the β chain and delay of internalization of the Mut.24/IL-2R complex, while increasing the reliance on the α chain of the receptor complex to increase binding affinity.







**B**

**A**

**Supplementary Figure 4 | Ratios of cell types on Day 4 after Fc.Mut24 or PBS treatment.** Frequency of CD4^+^CD25^+^Foxp3^+^ Tregs cells in **(A)** peripheral blood mononuclear cells and **(B)** splenocytes as a ratio to frequency of CD8a^+^ cells or CD11b^+^ cells. Data is presented as means with standard deviation.
